# Oleum Terebinthinæ in Acute Affections of the Middle Ear

**Published:** 1873-03

**Authors:** Fredr. Eugene Weber


### OLEUM TEREBINTHINÆ IN ACUTE AFFECTIONS OF THE MIDDLE EAR.

BY FREDR. EUGENE WEBER (*Monatschrift f. Ohrenhlkde Jahrg., V, No.* 3.)

*Translated from the Rundschau by J F. Noyes, M.D., Professor of Ophthalmology and Otology in Detroit Medical College.*

The writer remarks that for two years he has made use of the above-named remedy in acute and painful affections of the middle ear. His experience with it goes to show that there is no treatment, no medicinal preparation, which in so painful inflammation of the middle ear gives such immediate relief as the oil of turpentine.

Weber orders the medicine in every stage of acute otitis media so long as the pain continues. Leeches, cold applications, warm injections, etc., may be set aside while using the remedy internally, although in very severe cases they may aid in giving relief. The air douche, with the hand-bag, however, and Gruber's method of removing accumulated secretions, as also, if the drum is perforated, the careful cleaning of the outer and middle ear by means of tepid injections, are not to be dispensed with.

The writer recommends this remedy in relatively large doses. A small dose twice daily, and in the evening a full tablespoonful of oleum terebinthinæ, followed by lemon juice in the mouth, or, twice daily, capsules filled with this remedy, three of them in the morning, and five to six of them in the evening. In not a few patients it produces nausea, and, generally, dizziness occurs after taking. In such a case the morning dose may be lessened or diminished, but not the evening dose.

In inflammation of the middle ear, which may be complicated with nasal and throat catarrh, this remedy will accomplish less than in other cases. In the first instance, the writer directs the use of inhalations of water steam (flor. chamomile, with some oleoso-balsam, with boiling water poured upon it.)

After very severe operations upon the middle ear, the writer recommends the oil of turpentine as a prophylactic against inflammation, with the greatest confidence.

With this communication the writer appends the history of the following striking case last treated by him:

A young, very debilitated lady had suffered for four weeks with uninterrupted otalgia of the right side. At night the pain was intolerable, and had for nearly a month throughout rendered all sleep impossible. The attack, which was brought on by taking cold in a draft of air, and in the commencement was ushered in with pain in the throat, was followed by constant noise and singing in the ear. Later it was accompanied, also, by pain in the external ear, perhaps in consequence of too frequent instillation of warm drops and anodyne medicinal solutions.

The last remedy, also warm cataplasms, blistering behind the ears, leeching, recommended by other physicians, and, finally, the internal use of cinchona, opium, iodide potassium, were without benefit.

The objective examination gave an entirely negative result; so it was that the diagnosis otalgia nervosa was fixed upon. Weber ordered turpentine capsules in this wise, that on the same morning three, afternoon three, and in the evening six capsules were to be given. The ear was simply stopped up with charpie. The next day the patient reported that she had slept for the first time since four weeks; but that, after taking the turpentine, she was very much nauseated, and that after the six capsules she felt very dizzy. On the next day the patient took twice a day three capsules of oil of turpentine, each containing from four to twenty drops; in the evening four capsules. A few days after, the patient stated that the pain and noise in the ear were entirely gone, and that she in the night had slept well. During these days the patient took two capsules three times a day, and slept very well at night.

The pain and noise in the ear have not returned. Now the medication was stopped, as there was no further call for it, as, also, in the meantime, some strangury and diarrhœa had appeared.—*Detroit Review of Medicine and Pharmacy.*

